# Common Bean Subtelomeres Are Hot Spots of Recombination and Favor Resistance Gene Evolution

**DOI:** 10.3389/fpls.2018.01185

**Published:** 2018-08-14

**Authors:** Nicolas W. G. Chen, Vincent Thareau, Tiago Ribeiro, Ghislaine Magdelenat, Tom Ashfield, Roger W. Innes, Andrea Pedrosa-Harand, Valérie Geffroy

**Affiliations:** ^1^Institute of Plant Sciences Paris-Saclay (IPS2), UMR 9213/UMR1403, CNRS, INRA, Université Paris-Sud, Université d’Evry, Université Paris-Diderot Sorbonne Paris Cité, Orsay, France; ^2^IRHS, INRA, AGROCAMPUS OUEST, Université d’Angers, SFR 4207 QUASAV, Beaucouzé, France; ^3^Laboratory of Plant Cytogenetics, Federal University of Pernambuco, Recife, Brazil; ^4^Genoscope/Commissariat à l’Energie Atomique-Centre National de Séquençage, Evry, France; ^5^Department of Biology, Indiana University, Bloomington, IN, United States

**Keywords:** *Phaseolus vulgaris* (common bean), subtelomere, resistance gene, segmental duplication, evolution, satellite, homologous recombination (HR), non-homologous end-joining (NHEJ)

## Abstract

Subtelomeres of most eukaryotes contain fast-evolving genes usually involved in adaptive processes. In common bean (*Phaseolus vulgaris*), the *Co-2* anthracnose resistance (*R*) locus corresponds to a cluster of nucleotide-binding-site leucine-rich-repeat (NL) encoding sequences, the prevalent class of plant *R* genes. To study the recent evolution of this *R* gene cluster, we used a combination of sequence, genetic and cytogenetic comparative analyses between common bean genotypes from two distinct gene pools (Andean and Mesoamerican) that diverged 0.165 million years ago. *Co-2* is a large subtelomeric cluster on chromosome 11 comprising from 32 (Mesoamerican) to 52 (Andean) NL sequences embedded within *khipu* satellite repeats. Since the recent split between Andean and Mesoamerican gene pools, the *Co-2* cluster has experienced numerous gene-pool specific NL losses, leading to distinct NL repertoires. The high proportion of solo-LTR retrotransposons indicates that the *Co-2* cluster is located in a hot spot of unequal intra-strand homologous recombination. Furthermore, we observe large segmental duplications involving both Non-Homologous End Joining and Homologous Recombination double-strand break repair pathways. Finally, the identification of a Mesoamerican-specific subtelomeric sequence reveals frequent interchromosomal recombinations between common bean subtelomeres. Altogether, our results highlight that common bean subtelomeres are hot spots of recombination and favor the rapid evolution of *R* genes. We propose that chromosome ends could act as *R* gene incubators in many plant genomes.

## Introduction

Plant immunity is activated after direct or indirect perception of pathogen molecules by resistance (*R*) genes (Jones and Dangl, 2006; Kourelis and van der Hoorn, 2018). The proteins encoded by the largest class of plant *R* genes contain nucleotide binding site (NB) and leucine rich repeat (LRR) domains, together with a N terminus that usually contains a predicted coiled-coil structure (CC domain) or shares similarity with Drosophila Toll/mammalian interleukin 1 receptors (TIR domain). Genes encoding CC-NB-LRR (CNL) and TIR-NB-LRR (TNL) proteins correspond to two ancient lineages (Bai et al., 2002; Meyers et al., 2003; Ameline-Torregrosa et al., 2008) and are often organized in clusters of two to dozens of copies evolving at different rates (Kuang, 2004; Smith et al., 2004; Bresson et al., 2011; Ratnaparkhe et al., 2011; Luo et al., 2012; Baggs et al., 2017). Clustering of duplicated *R* genes, as observed in numerous plant species (Pryor and Ellis, 1993; Crute and Pink, 1996), has been suggested to favor *R* gene evolution against an ever changing array of pathogens (Hulbert et al., 2001). Indeed, after duplication, mutations occurring in paralogs and/or subsequent recombination between clustered genes can give rise to novel *R* gene specificities (Sudupak et al., 1993; Richter et al., 1995; McDowell, 1998; Meyers et al., 1998; Chin et al., 2001; McDowell and Simon, 2006, 2008; Kim et al., 2017). However, little is known about the dynamics of *R* gene clusters on a short (infra-species) time scale (Kuang, 2004; Zhou et al., 2007).

Common bean (*Phaseolus vulgaris*) is a major food crop in many areas of the Americas, Europe, Africa and Asia that supports the health and income of ∼400 million people in eastern Africa and ∼250 million in Central and South America. Two major gene pools have been identified for cultivated common bean, Andean (South America) and Mesoamerican (Mexico and Central America) (Broughton et al., 2003). These two gene pools diverged ∼0.165 million years ago (Mya) (Mamidi et al., 2013; Schmutz et al., 2014). In the common bean genome, most of the large *R* gene loci map at the end of linkage groups (LG) (Meziadi et al., 2016). This is the case for the *Co-2* and *B4 R* loci, localized at one end of LG-B11 and LG-B4, respectively (David et al., 2009; Chen et al., 2010). Many specific *R* genes and Quantitative Trait Loci (QTL) conferring resistance to a diverse selection of pathogens, including fungi and bacteria, have been mapped to these two loci (Creusot et al., 1999; Geffroy et al., 1999, 2000, 2008; Miklas et al., 2006; David et al., 2008; Chen et al., 2010). Sequencing of ∼0.95 Mb of the *Co-2* locus in an Andean genotype (G19833) revealed that it corresponds to an ancestral CNL cluster syntenic to the *Rpg1 R* cluster in soybean (Ashfield et al., 2004; Innes et al., 2008). Comparative genomics analyses revealed that a CNL sequence from the *Co-2* cluster has migrated to the *B4* locus through an ectopic recombination event between non-homologous chromosomes ∼20–54 Mya (David et al., 2009). Strikingly, in soybean this relocated CNL sequence has been pseudogenized, while in common bean it underwent an impressive amplification during the last 20 million years, leading to a *Phaseolus*-specific CNL subfamily comprising more than 29 members clustered within the *B4 R* cluster. We have hypothesized that the different fates of this CNL sequence in soybean (pseudogenization) and common bean (amplification) was due to divergent genomic environments (David et al., 2009; Geffroy et al., 2009). Indeed, in soybean the relocated CNL was pseudogenized in a euchromatin region, while in common bean the *B4* cluster arose in a region close to the telomere, called the subtelomere.

Subtelomeres, regions proximal to telomeres, are transition regions between the telomere and the chromosome-specific DNA sequence. Subtelomeres are difficult to define, varying in length from 20 kb in some yeast strains to several 100 kb in higher eukaryotes (Mefford and Trask, 2002; Brown et al., 2010). Sequence assembling of these regions is particularly challenging because they often contain a high density of repeated sequences (Britten, 1998; Eichler, 2001; Kellis et al., 2003). Thus, subtelomeres are often lacking from so-called whole-genome sequences and remain relatively understudied, especially in plants (Heacock et al., 2004; Kuo et al., 2006; Koukalova et al., 2010). Tandemly organized repeats, known as satellites, have been identified by cytogenetic analyses in the subtelomeres of several plants species (Evtushenko et al., 2010; Koukalova et al., 2010). In other kingdoms, subtelomeres are often viewed as variable loci containing fast-evolving gene families involved in adaptative processes (Brown et al., 2010). Moreover, some plant genomes such as maize, Arabidopsis, or rice contain blocks of heterochromatin, known as “knobs,” containing highly repeated satellite DNA (Mcclintock, 1929; Fransz et al., 2000; Cheng et al., 2001). Interestingly, most common bean subtelomeres bear terminal heterochromatic knobs. Fluorescent *In Situ* Hybridization (FISH) analyses revealed that the *B4 R* gene cluster is localized adjacent to heterochromatic knobs and associated with a *Phaseolus*-specific, 528-bp subtelomeric satellite repeat named *khipu*. The *khipu* satellite is found at most common bean terminal knobs, indicating frequent exchanges between common bean subtelomeric regions (David et al., 2009; Richard et al., 2013).

Given the distal localization of the *Co-2 R* gene cluster at one end of LG-B11, we wanted to further characterize the organization of this cluster and to understand its recent evolution since the divergence of Andean and Mesoamerican gene pools (0.165 Mya). To this end, we have extended the sequence of the *Co-2* cluster from ∼0.95 Mb to ∼1.35 Mb in G19833 (Andean) and sequenced ∼1.1 Mb in BAT93 (Mesoamerican). We then used a combination of comparative genomics approaches including computational analyses and FISH to study the recent molecular evolution of this locus. Altogether, our results highlight that subtelomeres are hot spots of intra- and inter-chromosomal recombination and favor the rapid evolution of *R* genes.

## Materials and Methods

### Bacterial Artificial Chromosome (BAC) Contig Assembly and Sequencing

To extend the G19833 BAC contigs previously sequenced in Innes et al. (2008), we selected five additional overlapping BAC clones based on G19833 BAC end sequence (BES) library (Schlueter et al., 2008). To identify the corresponding region in a Mesoamerican common bean genotype (BAT93), we screened a BAT93 BAC library (Kami et al., 2006) using DNA hybridization probes derived from low-copy protein-coding genes identified in the soybean *Rpg1* locus sequence (Innes et al., 2008). BAC clones that hybridized to two or more probes were fingerprinted and end-sequenced. Identification of a minimum-tiling path for sequencing was done as described in Innes et al. (2008). Sequencing and assembly were performed at Genoscope (Evry, France). Sequenced BAC clones and contigs are listed in **Supplementary Table S1**.

### Annotation Procedure

Gene prediction was done using a combination of gene-finding programs and sequence homology with known genes and proteins. Two *ab initio* gene prediction programs FGENESH (Burset and Guigó, 1996) and GeneMarkhmm (Lukashin and Borodovsky, 1998) were used. BLAST (Altschul et al., 1997) analyses against the GenBank non-redundant database were performed and the *Phaseolus* Expressed Sequence Tags (EST) available at GenBank (Ramirez et al., 2005) were aligned to the predicted genes as described in Samson et al. (2004). The criteria used to define a gene when there was no EST support were, first, a match to a sequence in a protein database using BLASTX (of 1e^-3^) on the non-redundant protein sequence database (nr) and, second, prediction as a gene by the two prediction programs. All this information was imported into the annotation platform Artemis for further manual analysis (Rutherford et al., 2000). Sequences were considered as pseudogenes when starting with a methionine, but presenting premature stop codons and/or frameshifts. Numerous sequences presenting homology to CNL sequences but that did not start with a methionine were retrieved and annotated as CNL segments.

### Identifying Retroelements, Repeats, and Segmental Duplications

Repetitive elements and transposon sequences were identified using BLASTN (of 1e-10) on the *P. vulgaris* repeat database (Gao et al., 2014), followed by manual inspection. BLASTN results corresponding to short degenerated sequences (<1000 bp) were discarded from the analysis. The program LTR_STRUC was used as the first step in identifying Long Terminal Repeats (LTRs) retrotransposon sequences (McCarthy and McDonald, 2003). Then, LTRs from the elements identified by LTR-STRUC, plus 134 LTRs previously identified in *Glycine* and *Phaseolus* genomes (Wawrzynski et al., 2008) were used as queries in BLASTN searches with a cutoff value of 1e-10 (Altschul et al., 1997). LTRs were classified as described in Wawrzynski et al. (2008). In order to optimize LTR finding, we performed a second BLASTN search using the entire dataset of previously identified LTRs. Regions of homology to known retrotransposon-like sequences (e.g., reverse transcriptase, integrase, etc.) were then manually evaluated for the presence of LTRs and Target Site Duplications (TSDs). These additional searches uncovered several intact elements missed by the LTR_STRUC program as well as solo-LTRs (**Supplementary Table S9**). The *khipu* satellite DNA was uncovered using hmmsearch “http://hmmer.janelia.org” with a *khipu* profile previously defined on 92 *khipu* units as explained in David et al. (2009). Segmental duplications were searched by BLASTN analyses within and between sequenced contigs from each genotype after masking *khipu* repeats and CNL genes, using the criterion of presenting more than 90% nucleotide identity over 500 bp minimum.

### Microsynteny Analysis

After annotation, we identified low-copy genes as genes presenting less than 20 hits in the Arabidopsis genome after BLASTP (of 1^e-5^) analysis using the FLAGdb^++^ database (Samson et al., 2004). Low-copy genes from BAT93 and G19833 genotypes were used for aligning the sequenced contigs. Orientation of G19833 contigs, previously aligned to the soybean and *Medicago truncatula* genomes (Innes et al., 2008), was confirmed by BLASTN analyses on the available G19833 genome (Schmutz et al., 2014) and used as reference for the orientation of BAT93 contigs (**Supplementary Table S1**). Twelve pairs of non-pseudogenized, non-truncated low-copy genes (**Supplementary Table S4**) were aligned with MAFFT version 6 using the L-INS-I strategy (Katoh et al., 2005). Ks values were determined using DNAsp version 5 using default parameters (Librado and Rozas, 2009).

### Phylogenetic Analysis of NL Sequences

**FIGURE 1:**
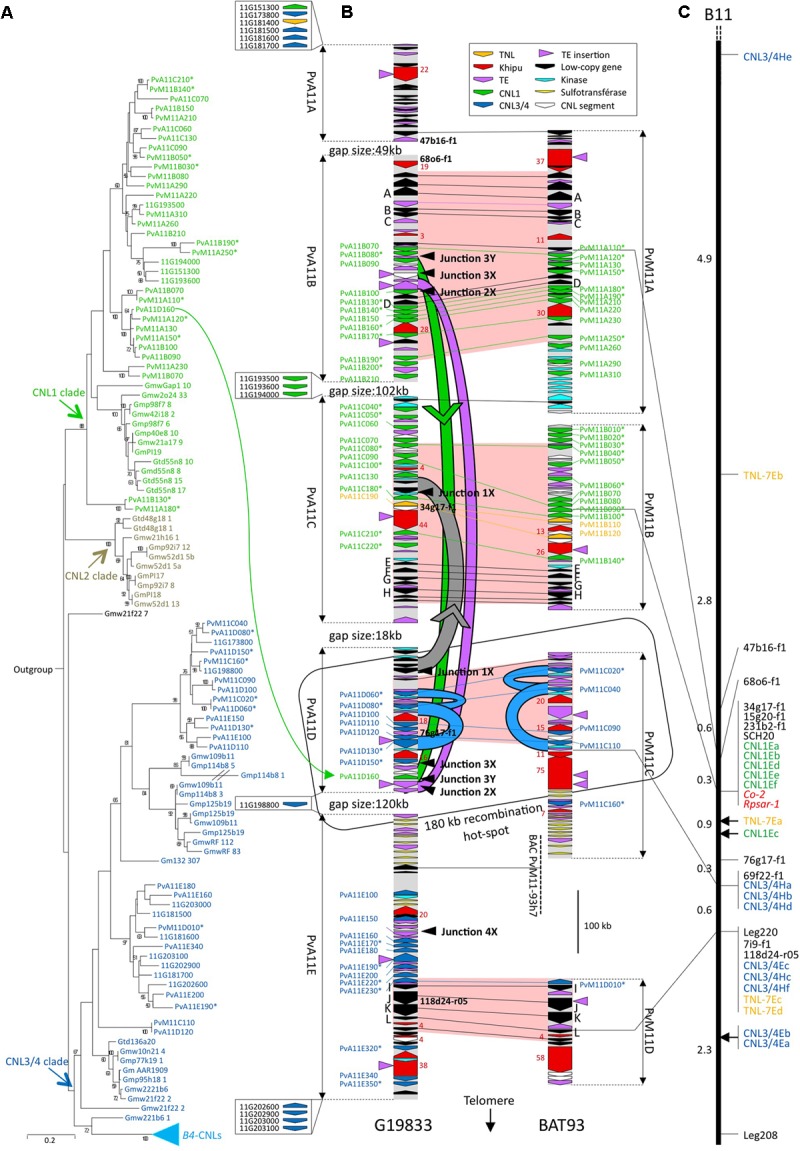
Phylogenetic analysis, physical and genetic maps of CNL genes. **(A)** ML tree of CNLs of the *Co-2* region. This tree was constructed using the CNL NB domains (from the P-loop to the MHD motif). Numbers at nodes indicate posterior probabilities (only > 50% are indicated). CNL names are colored according to major CNL1 (green) CNL2 (gray) and CNL3/4 (blue) clades. Gene names are abbreviated as follows: Gm, *G. max*; Gt, *G. tomentella*; PvA (or 11G when only found in WGS sequence) *P. vulgaris* Andean accession G19833; PvM, *P. vulgaris* Mesoamerican accession BAT93. **(B)** Physical map of the *Co-2* region in G19833 (Andean) and BAT93 (Mesoamerican) common bean genotypes. Arrows represent predicted genes, CNL segments, or *khipu* blocks and their orientation. CNL names and conserved low-copy genes (A to L) are listed on the flanks. NL retrieved outside sequenced contigs in G19833 WGS sequence are represented by arrows in boxes with corresponding names, to the left of G19833 sequenced contigs (not drawn to scale). Markers used for genetic mapping (black) and number of *khipu* repeats in a block (red) are listed in the inside. Fill colors of CNL genes correspond to the colors of the clades defined in **(A)**. Horizontal lines represent alleles of low-copy (black), CNL1 (green), CNL3/4 (blue) or TNL (yellow) genes. Red-filled zones correspond to regions strictly collinear based on low-copy and CNL alleles. Curved green, gray, or blue lines represent segmental green, gray, or blue duplication events, respectively. Green and gray arrowheads indicate the putative orientation of green and gray event, respectively. The large orange arrow represents the 34 kb SD from the *Co-2* cluster to chromosome 10. Black arrowheads at the right side of G19833 contigs point to corresponding junctions at the edge of segmental duplications. These junctions are detailed in **Figure 4**. **(C)** Genetic map of the *Co-2* locus (LG B11). Markers are listed on the right. Green and blue text correspond to the CNL1 and CNL3/4, respectively. Red text corresponds to resistance genes. Genetic distances between each marker are indicated on the left in Kosambi cM. An asterisk has been added at the end of CNL sequence names corresponding to pseudogenes.

Multiple alignments of amino acid sequences from the NB portion of CNLs or TNLs (from the P-loop to the MHD motif) were performed with MAFFT version 6 using the L-INS-I strategy (Katoh et al., 2005) with default parameters. Aligned protein sequences were used as a guide to align the corresponding DNA sequences. Recombination among loci was assessed using several methods implemented in RDP version 3.15 (Martin et al., 2010): RDP (Martin and Rybicki, 2000), Geneconv (Padidam et al., 1999), Chimera (Posada and Crandall, 2001), and Bootscan (Martin et al., 2005) as described in Ashfield et al. (2012). For CNLs, the alignment was then trimmed to an approximately 166 amino acid region of the NB domain devoid of recombination events. Alignments were then subjected to maximum likelihood (ML) analysis using the JTT model as implemented in PhyML (Guindon and Gascuel, 2003). Relative support for clades was assessed with 1,000 bootstrap replicates. The resulting phylogenetic trees (**Figure 1A** and **Supplementary Figure S2**) were displayed using MEGA 3.1 (Tamura et al., 2007). The TNL tree (**Supplementary Figure S2**) comprises the TNL sequences from the *Co-2* cluster (this study) and a diversity of common bean TNL sequences (Lopez et al., 2003), plus TNLs from the soybean *Rpg1* cluster (Innes et al., 2008) and TNLs from the whole genome of *Medicago truncatula* (Ameline-Torregrosa et al., 2008). Arabidopsis *TAO1* gene was used as nearest outgroup (Eitas et al., 2008). The CNL tree (**Figure 1A**) comprises sequences from the *Co-2* cluster, the soybean and *Glycine tomentella Co-2* syntenic clusters (Innes et al., 2008; Chen et al., 2010) as well as sequences from the *B4* cluster (David et al., 2009). The two closest CNLs from the *Co-2* family in the *M. truncatula* genome were used as nearest outgroup (Innes et al., 2008). CNL sequences with an intact and non-recombinant NB domain were grouped into subfamilies CNL1 or CNL3/4 (Chen et al., 2010) using phylogeny (**Figure 1A**). Whole CNL sequences were aligned using Needle (Needleman and Wunsch, 1970) and pseudogenes and/or recombinants were assigned to subfamilies CNL1 or CNL3/4 using a nucleotide identity threshold of 72% (**Supplementary Table S7**). Ks were determined on whole CNL alignments using DNAsp version 5 with default parameters (Librado and Rozas, 2009).

### Genetic Mapping

A PCR*-*based approach was used to map BAT93 contigs using specific oligonucleotide primers. For TNL mapping, a NB-specific 496-bp probe (**Supplementary Figure S5**) was amplified from TNL sequence PvM11B110 from BAC clone Pva1-231b2 using primers M231b2_3S (5′-TGGTTGCTTCCTCACAAATG-3′) and M231b2_3A (5′-TTCACTTTCCCATGCCTCTT-3′). This TNL probe was used in Southern Blot hybridization experiments as described in Geffroy et al. (1998). Chi-squared (χ^2^) tests were used to evaluate the goodness of fit of observed and expected segregation ratios. MAPMAKER software version 3.0 (Lander et al., 1987) was used to map segregating markers as described in Geffroy et al. (2000). All markers used in this study are described in **Supplementary Table S10** and were mapped using a population of 179 Recombinant Inbred Lines (RILs) derived from a cross between BAT93 (Mesoamerican) and JaloEEP558 (Andean) (Chen et al., 2010).

### Cytogenetic Analysis

DNA probes used in FISH experiments are described in **Supplementary Table S8**. FISH-CNL1, FISH-CNL3/4 and FISH-TNL probes correspond to pools of two to seven BAC subclones of ∼6 kb each, coming from sequenced BACs of the Andean genotype G19833. These probes contain NL sequences as well as few intergenic sequences (**Supplementary Figure S5**). Therefore, subclones were selected for the absence of repeats by performing BLASTX or BLASTN analyses of the intergenic portions of these subclones against (*i*) the GenBank non-redundant database, (*ii*) G19833 WGS sequence, (*iii*) a library of common bean repeat sequences based on 89,017 BES from G19833, and (*iv*) 1,404 shotgun sequences from the BAT7 Mesoamerican genotype (Schlueter et al., 2008; Schmutz et al., 2014). Moreover, to select against local repeats, BLASTN analyses were performed against the entire set of BAC-sequenced contigs from G19833 and BAT93. The *khipu* probe was generated using a pool of five BAC subclones comprising three BAC subclones coming from different *khipu* blocks spread over the sequenced contigs from the *Co-2* cluster, plus one additional subclone from another subtelomeric BAC from the short arm of chromosome 5 (Chen et al., 2010), and the 1H04 subclone from chromosome 4 (David et al., 2009). The 45S rDNA probe corresponds to the R2 probe, a 6.5-kb fragment of an 18S–5.8S–25S rDNA repeat unit from Arabidopsis (Wanzenböck et al., 1997). Pachytene chromosomes were prepared from young flower buds fixed in ethanol:acetic acid (3:1, v/v). Buds were macerated in 2% cellulase/2% pectolyase/2% cytohelicase in 0.01 M citric acid-sodium citrate buffer, pH 4.8, for 3 hr at 37°C, incubated in 60% acetic acid up to 2 h, and squashed after removal of petals and sepals and flaming. Slide selection and pretreatment, chromosome and probe denaturation and hybridization, posthybridization washes, detection and image analyses were performed as described in Fonsêca et al. (2010).

## Results

### Sequencing and Annotation of the *Co-2* Resistance Locus in Two Common Bean Genotypes

In a common bean genotype of Andean origin (G19833), we have previously sequenced, annotated and mapped five BAC contigs corresponding to a 0.95-Mb region centered on the *Co-2 R* locus (Innes et al., 2008; Chen et al., 2010). Here, we sequenced five additional overlapping BAC clones, leading to a total of 1.35 Mb organized in five contigs (PvA11A to PvA11E, **Figure 1B** and **Supplementary Table S1**); however, the four gaps between the contigs remained unclosed. We have compared our BAC-based sequence data with the whole genome shotgun (WGS) sequencing data of G19833 for this region (Schmutz et al., 2014). The five contigs were located at the end of chromosome 11 long arm of the WGS sequence, and the four gaps were found to be 48833, 101827, 18552, and 120389 bp, respectively (**Supplementary Table S1** and **Figure 1B**).

In order to study the evolution of the *Co-2 R*-gene cluster since the divergence between Andean and Mesoamerican gene pools, we selected and sequenced nine BACs from a Mesoamerican common bean genotype (BAT93), leading to 1.07 Mb organized in four contigs (PvM11A to PvM11D, **Figure 1B**). PCR-based mapping confirms that macrosynteny between G19833 and BAT93 contigs is consistent with microsynteny (**Figures 1B,C**). Automatic and manually curated sequence annotation predicted 116 genes and pseudogenes in the G19833 sequence and 90 genes and pseudogenes in the BAT93 sequence. For both genotypes, the genic part represents one third of the total sequence, which corresponds to an average density of one gene per 12 kb (**Supplementary Table S2** and **Supplementary Figure S1**). The overall organization of the *Co-2* locus consists of low-copy gene islands separated by numerous genes from the CNL, kinase and sulfotransferase multigenic families, transposable elements, and the *khipu* 528-bp subtelomeric satellite (David et al., 2009; **Figure 1B**). These numerous repeated sequences impacted the quality of the WGS sequence in G19833. As a result, 76 gaps corresponding to 167 kb were missing in the WGS sequence corresponding to our 1.35 Mb BAC-based sequences.

CNL sequences from the *Co-2* family represent around one third of the predicted genes in both genotypes, with 43 CNLs in G19833 and 30 CNLs in BAT93 (**Figure 1B** and **Supplementary Figure S1**). For G19833, most CNL sequences were 100% identical between the WGS and our BAC-based sequence. However, 10 CNLs were either missing or contained errors in the WGS while 13 additional CNLs were retrieved in the WGS sequence outside our BAC contigs, leading to a total number of 56 CNLs in G19833 (**Supplementary Table S3**).

The CNL sequences from the *Co-2* cluster were previously classified into four subfamilies (referred to as CNL1 to CNL4) that diverged prior to the split between common bean and soybean, 20 Mya (Lavin et al., 2005; Innes et al., 2008; Chen et al., 2010). CNL2 sequences were not found in the common bean genome, indicating that these sequences were specifically lost in common bean (Chen et al., 2010). We refer to CNL3/4 to describe CNL3 plus CNL4 sequences, because CNL3- and CNL4-specific probes cross-hybridize on common bean DNA (Chen et al., 2010). As previously shown in Innes et al. (2008), we observed two well-defined blocks of proximal CNL1 and distal CNL3/4 sequences, suggesting that these CNL copies arose mainly by tandem duplications.

Interestingly, in addition to the prevalent CNL sequences, we also identified one TNL sequence in G19833, collinear to two tandemly duplicated TNL sequences in BAT93, and an additional TNL sequence was retrieved from G19833 WGS annotation (in yellow on **Figure 1B**). Phylogenetic analyses showed that these TNLs belong to the *M. truncatula* TNL-7 subfamily defined in Ameline-Torregrosa et al. (2008) (**Supplementary Figure S2**). In total, the number of NL sequences at the *Co-2* locus is 58 (56 CNL plus 2 TNL) in G19833 and 32 (30 CNL plus 2 TNL) in BAT93. Notably, in G19833 52 NL sequences are clustered within 1,35 Mbp (**Supplementary Table S3**). Consequently, *Co-2* is one of the largest NL clusters identified to date in plants, together with the maize *Rp1* cluster (Smith et al., 2004).

### The *Co-2* Cluster Arose From CNL Duplications That Occurred 20 Mya to 0.165 Mya Ago, Followed by Gene-Pool Specific CNL Deletions and Pseudogenization

The nucleotide substitution rate at silent sites (Ks) is a measure of neutral evolution for coding DNA because it doesn’t take in account the non-synonymous sites that can be under selection pressure. Therefore, Ks is often used to estimate the time of divergence between two similar genes, these genes being either orthologs (for dating the divergence between taxa) or paralogs (for dating a duplication event). Lower *K*s values correspond to shorter times of divergence between sequences. Here, we wanted to know if the duplication of CNL paralogs from the *Co-2* cluster occurred before or after the divergence between Andean and Mesoamerican gene pools, 0.165 Mya. To this end, we used the Ks from 12 ortholog pairs of low-copy genes as reference to infer orthology and paralogy relationships between CNL sequences (**Figure 1B** and **Supplementary Table S4**). The mean *K*s value for these 12 ortholog pairs is 0.0177, with a maximum of 0.0494. Thus, we consider that a *K*s below 0.0494 is indicative of events that are either simultaneous or more recent than 0.165 Mya.

Between gene pools, 16 CNL pairs have *K*s values under 0.0494 and can thus be considered as orthologs (**Figure 1B** and **Supplementary Tables S5, S6**). Consistently, all these orthologs are found in syntenic positions (**Figure 1B**) and share more than 96% nucleotide identity within a pair while all other CNL sequences share less than 95% identity with each other (**Supplementary Table S7**). These 16 CNL pairs comprise 11 intact full-length genes and 21 pseudogenes (i.e., CNLs presenting frameshifts and/or truncated before the usual end of CNL coding sequence). As a result, only three pairs are composed of intact CNL genes in both genotypes while one and four CNLs are specifically intact in G19833 and BAT93, respectively. Manual inspection indicated that only four pseudogenization events were common to both members of a pair and consequently occurred before 0.165 Mya, while 13 occurred more recently and independently in the Andean and Mesoamerican gene pools (**Supplementary Table S6**). Within each genotype, we detected only one pair of CNL paralogs with a *K*s value under 0.0494, thus corresponding to a recent duplication that occurred after the divergence of both gene pools (**Supplementary Tables S5, S6**). This recent event is specific for G19833 and corresponds to the ectopic duplication of PvA11B090 to PvA11D160 (green arrow in **Figure 1B**). Consequently, except for this case, all CNLs appear to have emerged by duplications predating the divergence between Andean and Mesoamerican gene pools.

Low-copy genes and CNL orthologs allowed us to delineate four collinear regions spanning 865 kb in total (red zones in **Figure 1B**). No recent paralogs were found within these regions, as testified by the *K*s values over 0.0494 for every paralog pair (**Supplementary Table S5**). Thus, presence of a CNL in only one genotype indicates that the corresponding ortholog has been lost in the other genotype. Following this rule, we identified six G19833-specific losses (corresponding to PvM11A120^∗^, PvM11A150^∗^, PvM11B050^∗^, PvM11B060^∗^, PvM11B070, PvM11B090^∗^ in BAT93) and three BAT93-specific losses (corresponding to PvA11C130, PvA11C220^∗^, PvA11D110 in G19833). In all, we identified nine recent CNL losses within the red zones.

In all, genomic comparison between one Andean and one Mesoamerican genotype revealed intensive evolution of the CNL sequences from the *Co-2* cluster within the last 0.165 My. In particular, we detected one gene pool-specific CNL duplication, nine gene pool-specific CNLs losses, and 13 pseudogenization events, indicating a strong erasure of CNLs from 0.165 Mya to the present day. As a result, only three intact CNLs are conserved in both the Andean and Mesoamerican genotypes.

### FISH Experiments Show Differentiations in the NL Repertoire of Mesoamerican Versus Andean Subtelomeres

**FIGURE 2:**
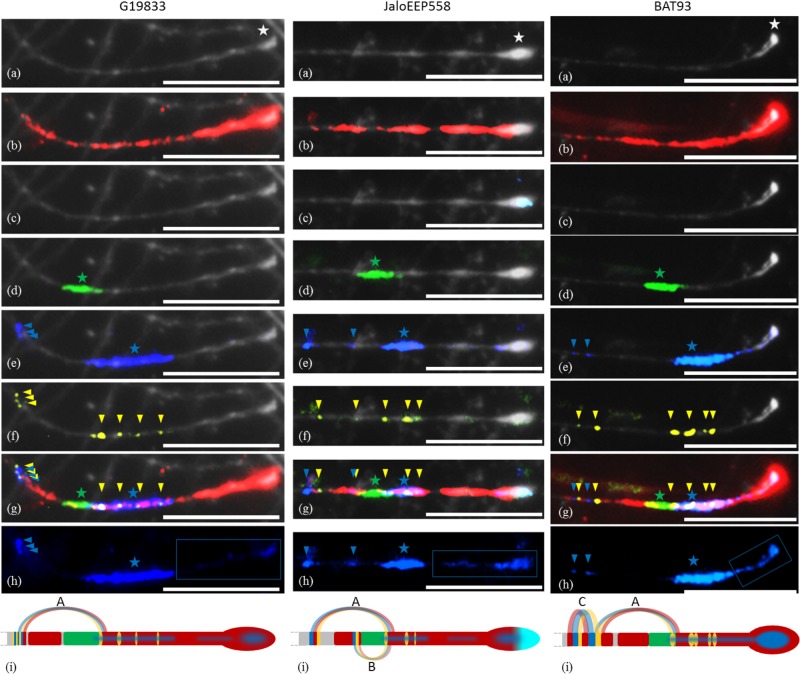
Pachytene FISH mapping of multiple probes at the end of chromosome 11 in common bean genotypes G19833 and JaloEEP558 (Andean) and BAT93 (Mesoamerican). These experiments were done at least twice for each genotype with similar results, except for G19833 because it was highly difficult to obtain buds containing cells at the pachytene stage from this genotype. The number of loci for each probe was confirmed in at least three cells per experiment. Bars = 10 *μ*m. **(a)** Black-white-converted image showing the end of chromosome 11 stained with DAPI. Terminal knob is indicated by a white star. **(b)** *khipu* satellite (red). **(c)** 45S rDNA (light blue). For G19833, cells were too much damaged for analyzing 45S rDNA. **(d)** FISH-CNL1 probe (green). The large CNL1 signal corresponding to the *Co-2* cluster is indicated by a green star. **(e)** FISH-CNL3/4 probe (blue). The large CNL3/4 signal corresponding to the *Co-2* cluster is indicated by a blue star. Additional CNL3/4 loci are indicated by blue arrowheads. **(f)** FISH-TNL-7 probe (yellow). Loci are indicated by yellow arrowheads. **(g)** Overview of FISH-CNL1 (green), FISH-CNL3/4 (blue), FISH-TNL-7 (yellow), khipu (red) and 45S rDNA (light blue). The main CNL1 and CNL3/4 clusters (corresponding to region sequenced in **Figure 1**) are indicated by a green star and a blue star, respectively. Additional CNL3/4 and TNL-7 loci are indicated by blue and yellow arrowheads, respectively. **(h)** FISH-CNL3/4 probe without DAPI counterstaining. The terminal FISH-CNL3/4 signals are boxed. The main CNL3/4 cluster is indicated by a blue star. Additional CNL3/4 loci are indicated by blue arrowheads. **(i)** Diagrammatic representation of cytogenetic maps of chromosome 11 long arm terminal region, and hypothetical model of evolution. Chromosomes are in gray, and colors are the same as described above. Curved lines represent minimal putative ectopic segmental duplications involving TNL-7 (yellow), CNL3/4 (blue), and *khipu* (red).

In order to determine the physical distribution of CNL1, CNL3/4 and TNL-7 sequences in the common bean genome, we used specific probes for FISH experiments on pachytene chromosomes from G19833 (Andean), JaloEEP558 (Andean) and BAT93 (Mesoamerican) genotypes (**Supplementary Table S8**). We also used the subtelomeric DNA satellite *khipu* (David et al., 2009) and the subtelomeric 45S rDNA (Pedrosa-Harand et al., 2006) as probes for labeling subtelomeric regions. In all genotypes, the end of chromosome 11 long arm is composed of a large terminal heterochromatic knob, followed by a region not uniformly condensed (**Figure 2a**). In agreement with the analysis of *khipu* sequences in G19833 WGS (Richard et al., 2013), chromosome 11 long arm is the genomic region presenting the largest *khipu* signal in the entire common bean genome. Here, *khipu* signal is similar in all genotypes, extending from the terminal knob chromosome 11 long arm to a large region proximal to the knob (**Figure 2b**). In JaloEEP558, the terminal knob is caped with 45S rDNA, while no 45S signal appears in the other genotypes (**Figure 2c**).

FISH-CNL1 and FISH-CNL3/4 probes show large signals overlapping with *khipu* (**Figures 2d,e**). These large signals likely correspond to the *Co-2* cluster. In BAT93, the *Co-2* cluster is located closer to the terminal knob than in the Andean genotypes. Furthermore, the FISH-CNL3/4 signal is wider in G19833 than in the two other genotypes, suggesting that specific duplications of CNL3/4 sequences occurred in this genotype. In contrast to CNL1 sequences that are localized at only one locus in the common bean genome, additional smaller FISH-CNL3/4 signals appear at positions proximal to the *Co-2* cluster, indicating that CNL3/4 sequences moved by ectopic duplications (**Figure 2e**). FISH-TNL-7 signals appear as spots either overlapping with or proximal to the *Co-2* cluster (**Figures 2f,g**), suggesting that TNL-7 sequences amplified more by ectopic duplication than tandem duplication. All these results are consistent with RFLP-based mapping showing that CNL1 sequences map only at one genetic position while CNL3/4 and TNL-7 sequences are distributed at both proximal and distal positions compared to CNL1 sequences (**Supplementary Figure S3**).

**FIGURE 3:**
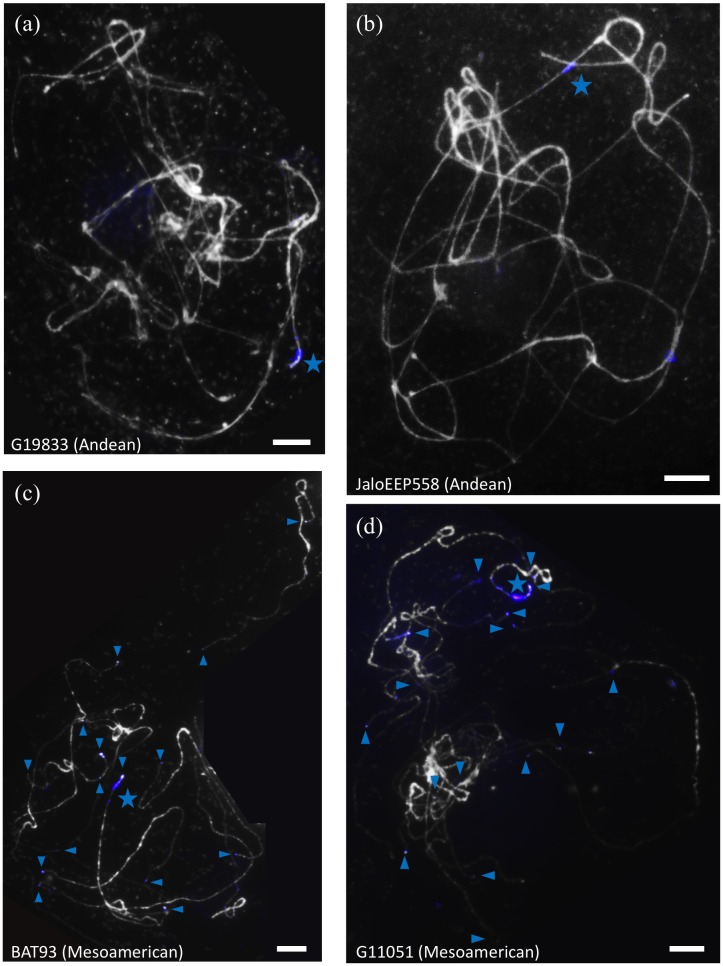
Localization of FISH-CNL3/4 probe on pachytene chromosomes of common bean genotypes from the Andean and Mesoamerican gene pools. Chromosomes are counterstained with DAPI (gray), and FISH-CNL3/4 signals are in blue. The *Co-2* locus is indicated by a blue star, and FISH-CNL3/4-labeled terminal knobs are indicated by blue arrowheads. **(a)** G19833 (Andean), **(b)** JaloEEP558 (Andean), **(c)** BAT93 (Mesoamerican), **(d)** G11051 (Mesoamerican). These experiments were done at least twice for each genotype with similar results. The number of loci for each probe was confirmed in at least three cells per experiment. Bars = 10 μm.

Unexpectedly, the terminal knob of chromosome 11 long arm was labeled not only with *khipu* but also with the FISH-CNL3/4 probe (**Figure 2h**). At the whole genome level, 14 terminal knobs were labeled with strong FISH-CNL3/4 signals in BAT93 (Mesoamerican), and 16 in another Mesoamerican genotype (G11051; **Figure 3**). This is in sharp contrast with Andean genotypes where only two and three very weak FISH-CNL3/4 signals were observed at JaloEEP558 and G19833 terminal knobs, respectively (**Figure 3**). These FISH signals likely corresponded to a portion of a CNL3/4 gene or to an intergenic portion of the subclones used for FISH-CNL3/4 probe (**Supplementary Figure S4**). Unfortunately, we were unable to retrieve these sequences in the WGS of G19833, because the pseudomolecules contain numerous gaps at their ends (Schmutz et al., 2014). Interestingly, these results suggest that a sequence linked to CNL3/4 genes has become a subtelomeric repeat that amplified much more in Mesoamerican than Andean genotypes, both locally (stronger signals) and between non-homologous chromosomes (multiple labeled terminal knobs).

Altogether, these results indicate that frequent local and inter-chromosomal recombination events occurred at common bean subtelomeres. In particular, gene pool specific ectopic duplications of CNL3/4 and TNL-7 sequences led to different NL repertoires in Mesoamerican versus Andean subtelomeres.

### Local and Ectopic Segmental Duplications Involving NL Sequences Have Occurred in Common Bean

Within the subtelomere of chromosome 11 long arm, spots of contiguous FISH-TNL-7, FISH-CNL3/4 and *khipu* signals at positions proximal to the main *Co-2* locus indicate that ectopic duplications involving TNL-7, CNL3/4, and *khipu* sequences occurred (**Figure 2g**). The signals from these three different probes are contiguous, suggesting that these ectopic duplications have been due to segmental duplications (SDs) involving TNL-7, CNL3/4, and *khipu* sequences. We summarize these hypothetical SD events as a model in **Figure 2i**. SD event A is observed for all genotypes, suggesting that it predates the divergence between Andean and Mesoamerican genotypes, while events B, and C likely occurred specifically in JaloEEP558 and BAT93, respectively. In G19833 WGS, in addition to the two full length TNL-7 present in the annotation, BLASTn analysis allowed us to retrieve four TNL-7 segments that were located in positions congruent with the FISH-TNL-7 signals (**Supplementary Figure S5**). This allows us to show that event A corresponds to the duplication of a couple of TNL-7 and CNL3/4 sequences in a tail to tail fashion (**Supplementary Figure S5d**). Two additional ectopic duplications involving these TNL-7 segments and CNL3/4 sequences occurred in G19833 (**Supplementary Figure S5d**). The intergenic portion between these TNL-7 and CNL3/4 sequences is partially conserved between duplicates, confirming that these sequences have been duplicated by SDs (data not shown). Interestingly, among the eight NL sequences found in these SDs, six were pseudogenized or interrupted by a TE insertion, leading to only one intact TNL-7 and one intact CNL3/4.

Within the BAC clone sequences, computational analysis allowed us to detect additional SDs in the *Co-2* cluster. We identified two ancient SDs shared by BAT93 and G19833 at different genomic positions (blue events in **Figure 1B**). These blue events correspond to the double duplication of a 10 kb region containing a CNL3/4 sequence and share ∼80% nucleotide identity between duplicated regions. Moreover, we identified three recent Andean-specific SDs defined as the green (22 kb), the purple (16 kb), and the gray (23 kb) SDs in G19833 (**Figure 1B**). The green SD resulted in the aforementioned duplication of PvA11B90 to PvA11D160 (**Figures 1A,B**). The absence of corresponding SDs in BAT93 indicates that these three events occurred less than 0.165 Mya, which is in agreement with the high nucleotide identity found between duplicated regions (>98%). Interestingly, all these SDs involved a region of only 180 kb, suggesting that this region is a hot-spot of recombination (**Figure 1B**). Moreover, the blue and green SDs contain intact CNL sequences (blue: PvMC040, PvMC110, PvA11D120; green: PvA11B090, and PvA11D160) suggesting that these SDs may have contributed to the duplication of functional *R* genes.

### Segmental Duplications Involve Both Homologous and Non-homologous Recombination Pathways at the *Co-2* Locus

**FIGURE 4:**
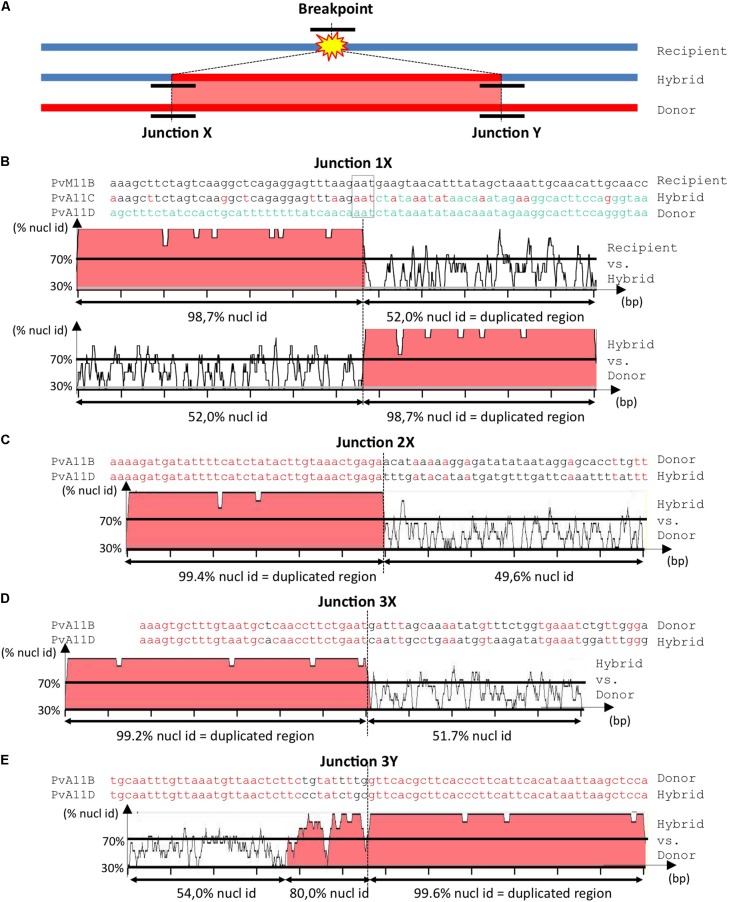
Analysis of junctions from SD‘ events at *Co-2* subtelomeric region of chromosome 11 long arm. **(A)** Simplified summary model describing the resulting stages of a SD event. The double-strand break occurs somewhere in the recipient (blue) molecule. Before repair, partial digestion of the breakpoint borders may occur, leading to loss of sequence. During the repair process, there is a duplicative invasion of the recipient molecule by the donor (red) molecule. Black short lines represent the regions that has to be aligned to accurately resolve the junctions (X and Y) between the broken molecule (recipient) and the duplicated region (donor), resulting in the final hybrid molecule. For more detailed information on double-strand break repair models, please refer to Puchta (2005) and Fiston-Lavier et al. (2007). **(B–E)** Nucleotide alignment at junctions 1X, 2X, 3X, and 3Y, respectively. Sequences are named according to their corresponding contigs. Aligned matching bases are red. For larger-scale vizualization, the 1000 bp surrounding region was analyzed using mVISTA (Mayor et al., 2000) after indels removal. Identity between sequences is indicated in %. Regions with identity equal or above 70% in 10bp windows are filled in red. Note that when the hybrid molecule is retained in the progeny, the recipient molecule is lost and a putative recipient molecule has to be found in another genotype to help resolving the breakpoint event. Therefore in **(B)** we used PvM11B contig from BAT93 genotype to help resolving junction 1X from an SD that occurred between PvA11B and PvA11C contigs in G19833 genotype. For junctions 2X **(C)**, 3X **(D)** and 3Y **(E)**, the recipient was not retrieved in BAT93, therefore the junctions are a bit less accurately resolved, but it was sufficient to infer the associated repair mechanism (i.e., NHEJ or HR).

Ectopic recombination can result from aberrant DNA repair by either Non-homologous End-Joining (NHEJ) or non-allelic homologous recombination (HR), which are two general pathways of double-strand break (DSB) repair in plants and other living organisms (Gorbunova and Levy, 1999; Lieber et al., 2003; Linardopoulou et al., 2005; Puchta, 2005; Pacher et al., 2007). In the case of SD, the broken recipient molecule is invaded at the breakpoint by an ectopic donor molecule, resulting in a hybrid molecule with two junctions (X and Y) corresponding to two repair points for each DSB (**Figure 4A**). Defining a breakpoint accurately requires comparison of the hybrid sequence to the original donor and recipient sequences. The presence of sequence similarity that extends beyond the homology boundary between duplicated regions is strongly suggestive of non-allelic HR, while the absence of transition or microhomology is indicative of NHEJ (Linardopoulou et al., 2005; Fiston-Lavier et al., 2007). Using these criteria, we deduced the probable repair process at five breakpoints from the green, purple, and gray SDs (**Figures 1B**, **4**).

For junction 1X, as the SD is G19833-specific, we retrieved the corresponding donor sequence in BAT93 (**Figure 1B**). Microhomology of only three identical nucleotides indicates that junction 1X was likely resolved by NHEJ (**Figure 4B**). This is illustrated by an abrupt drop in identity from more than 98% to around 50% between the hybrid and corresponding recipient and donor sequences. We used this later criterion for analyzing the putative repair pathways for the other breakpoints because we couldn’t find corresponding recipient sequences. Junctions 2X and 3X present the same identity percentage profile as junction 1X and were thus also resolved by NHEJ (**Figures 4C,D**). Conversely, junction 3Y was resolved by HR-mediated repair as testified by the presence of a 130 bp transition region with 80% nucleotidic identity between the hybrid and the donor sequence (**Figure 4E**). Junctions 3X and 3Y delineate a single SD indicating that a single SD could involve both HR and/or NHEJ pathways.

### Removal of LTR Retrotransposons by Homologous Recombination Is Occurring at a High Rate at the *Co-2* Locus

Within the BAC contig sequences, we identified 88 transposable elements in G19833 (1/15 kb) and 58 in BAT93 (1/18 kb), with a predominance of Long Terminal Repeats (LTR) retrotransposons in both G19833 (42) and BAT93 (41). Among them, 20 and 25 comprise identifiable LTR in G19833 and BAT93, respectively (**Supplementary Table S9**). The ratio between intact LTR retrotransposons and solo-LTRs (I/S ratio) is often used as an indicator of DNA removal caused by unequal HR (presumably intrastrand) between LTR sequences of an individual element (Devos et al., 2002; Ma and Bennetzen, 2004; Bennetzen et al., 2005; Vitte and Bennetzen, 2006; Sasaki et al., 2010; El Baidouri and Panaud, 2013). LTR sequence analysis revealed I/S ratios of 1.2 and 1.7 in BAT93 and G19833, respectively (**Supplementary Table S9**). These values are much lower than those calculated in the syntenic regions of soybean (8.0) and *Glycine tomentella* (7.7), which are not subtelomeric (Wawrzynski et al., 2008), indicating that at the *Co-2* locus, removal of LTR-retroelements via HR is 5 to 7 times more frequent in common bean than in closely related plant species. At the subtelomeric *B4* CNL cluster (David et al., 2009), we found an I/S ratio of 2, which is similar to the value from the *Co-2* cluster. Conversely, in a previous study of ∼1 Mbp located in a non-subtelomeric region of common bean chromosome 5, Lin et al. (2010) did not find any solo-LTRs for 17 intact LTR-retrotransposons, indicating that HR between LTRs is almost absent in this region. Together, these results indicate that both *Co-2* and *B4* clusters are highly dynamic in terms of HR, and that this property is not specific for the whole common bean genome, but rather specific for subtelomeres.

## Discussion

In various organisms (human, yeast, *Plasmodium)*, subtelomeres have been shown to have great potential for facilitating rapid adaptation because of their highly dynamic nature (Brown et al., 2010). Consequently, subtelomeres are ideal locations for genes that can benefit from this plasticity and they often bear larger and more rapidly evolving genes than those found at more internal chromosomal locations. However, little is known about plant subtelomeres and the genes present at these peculiar genomic locations. In the present study, we used a combination of genetics, cytogenetics, and computational approaches in Andean and Mesoamerican common bean genotypes to precisely characterize the recent molecular events that shaped the evolution of the *Co-2 R* cluster within a recent 0.165-My timescale The *Co-2* cluster is located at the subtelomere of chromosome 11 long arm, close to a heterochromatic terminal knob. This region is very peculiar as it contains the *khipu* satellite DNA repeats (Richard et al., 2013).

At the genome level, the presence of *khipu* satellite sequences at most terminal knobs combined with the fact that *khipu* is specific to the *Phaseolus* genus is evidence of intense interchromosomal shuffling between common bean subtelomeres (David et al., 2009; Geffroy et al., 2009; Richard et al., 2013). Here, FISH experiments unveiled a dramatic amplification of a novel repeat, linked to CNL3/4 sequences, at terminal knobs in Mesoamerican genotypes. These FISH signals could correspond to sequences associated to CNL3/4 in the intergenic portions of the subclones used as probes, or to a portion of a CNL3/4 gene. Sequencing BAT93 subtelomeres would help solve this puzzle. This result indicates that ectopic recombination between non-homologous subtelomeres has been sufficiently frequent to spread this repeat at most terminal knobs in less than 0.165 My. Such high plasticity at subtelomeric regions has not been reported so far in other plant species except in rye (González-García et al., 2006; Evtushenko et al., 2010). For example, subtelomeres from the model plant Arabidopsis present a simple organization (Kuo et al., 2006). Interestingly, the organization of common bean subtelomeres is very similar to human subtelomeres, which are hot-spots of interchromosomal recombination and SDs (Linardopoulou et al., 2005). Previous studies have shown that junctions from breakpoints occurring at subtelomeres were predominantly resolved by NHEJ, in Arabidopsis as well as in human, whatever the complexity of subtelomere organization (Linardopoulou et al., 2005; Kuo et al., 2006). Our results also suggest a predominance of NHEJ (three junctions) compared to HR (one junction) at common bean subtelomeres. In particular, a single SD could involve both HR and NHEJ pathways (junctions 3X and 3Y; **Figures 1, 4**) as it was previously observed in *Drosophila melanogaster* (Fiston-Lavier et al., 2007).

In plants and other organisms such as *Drosophila melanogaster* and human, the presence of repeated sequences and proximity to heterochromatin is thought to favor unequal recombination as well as SDs (Puchta, 2005; Fiston-Lavier et al., 2007; Kirsch et al., 2008). Here, the low LTR I/S ratios observed at the *Co-2* and *B4 R* clusters compared to I/S ratio observed for the syntenic region in soybean (Wawrzynski et al., 2008) or for non-subtelomeric region of common bean (Lin et al., 2010) suggest that common bean subtelomeres are highly dynamic in terms of unequal intrastrand HR. As proposed for other plant genomes, a high frequency of solo-LTRs sequences suggests that both the *Co-2* and *B4* clusters are contracting due to recombination events between homologous sequences (Devos et al., 2002; Ma and Bennetzen, 2004; Bennetzen et al., 2005; Vitte and Bennetzen, 2006; Ammiraju et al., 2008). In agreement with this, we observed numerous gene pool-specific losses of CNL at the *Co-2* cluster, suggesting that subsequent to an impressive CNL burst that occurred specifically in common bean compared to soybean (Innes et al., 2008; David et al., 2009; Ashfield et al., 2012), the *Co-2* cluster is now in the process of decay, in a gene pool specific manner. However, the erosion of the *Co-2* cluster has been counteracted by SDs, both locally and in ectopic regions, indicating that SDs played a major role in the recent NL evolution. Importantly, because our analysis is based on BAC clones, and because most of the SDs identified were located on different BAC clones, we can exclude out the fact that these SDs were due to assembly mistakes.

A significant finding, based on Ks analyses, was that numerous NL genes within the *Co-2* cluster have been specifically removed or pseudogenized in a gene pool specific manner while new NL sequences have been duplicated at ectopic regions, leading to continued amplification and diversification of the *Co-2* family. As a result, only three intact CNL genes were found in both G19833 and BAT93 while one and four CNLs were specifically intact in each genotype, indicating that Andean and Mesoamerican genotypes bear different NL repertoires. This suggests that most *R* specificities have diverged between these two gene pools. In agreement with this, previous cross-inoculation experiments between common bean plants and *Colletotrichum lindemuthianum* strains at the level of the centers of diversity of the plants indicated that ongoing processes of coevolution between the two protagonists have led to a differentiation for resistance (Geffroy et al., 1999).

With more than 52 NL sequences in G19833, the *Co-2* cluster is one of the largest *R* gene clusters identified so far in plants, together with the maize *Rp1* cluster (up to 52 NL sequences), the lettuce *RGC2* cluster (up to 32 NL sequences) and the bean *B4* cluster (more than 29 CNL sequences) (Kuang, 2004; Smith et al., 2004; David et al., 2009; Geffroy et al., 2009; Richard et al., 2017). The *Co-2* and *B4* clusters both belong to the common bean genome, and are both subtelomeric, suggesting that common bean subtelomeres favor *R* gene proliferation. We previously proposed that (*i*) a subtelomeric position and (*ii*) proximity to satellite repeats and heterochromatin could constitute a genomic environment favorable for *R* gene proliferation (David et al., 2009). This raises the question as to whether or not the large NL clusters from other plants are also subtelomeric. In agreement with this hypothesis, *RGC2* and *Rp1* clusters are both located at the very end of chromosomes, and a subtelomeric location was confirmed by FISH for *RGC2* in lettuce (Shen et al., 1998; McHale et al., 2009; Cheng et al., 2012). Analyses of NL distribution in various available plant genomes such as potato (Jupe et al., 2012), tomato (Wei et al., 2016), poplar (Kohler et al., 2008), grapewine (Velasco et al., 2007), Medicago (Ameline-Torregrosa et al., 2008; Young et al., 2011), and rice (Zhou et al., 2004), confirm that most large NL clusters are located at the end of the chromomes, suggesting a subtelomeric location even if not confirmed by FISH analysis. Altogether, these observations support the hypothesis that plant chromosome ends act as NL incubators leading to the production of large NL clusters, and favor the diversification of *R* genes.

## Conclusion

Our results highlight the role of SDs in NL evolution at subtelomeric regions and suggest that common bean subtelomeres constitute a genomic environment where elevated intra- and inter-chromosomal recombination frequencies provide a perfect niche to fuel rapid NL diversification. This is in agreement with what has been observed in other organisms (human, yeast, *Plasmodium*…) where subtelomeric regions present several unusual characteristics, such as the presence of repetitive DNA, increased rate of evolution and enrichment for genes involved in adaptation. However, plasticity of subtelomeres has rarely been reported in plants. In fact, many papers report that large NL clusters are located at chromosome ends in plants, and here we propose that subtelomeres could act as *R* gene incubators in plant genomes. It’s interesting to note that in some eukaryotic pathogens, genes for host-translocated effectors are also concentrated at chromosome ends (Kamoun, 2007; Chuma et al., 2011; Upson et al., 2018). These observations underline similarities between pathogens and hosts strategies for recruitment of special genomic niches that may accelerate the evolution of coevolving genes.

## Accessions Numbers

Sequencing data generated for this study have been submitted to GenBank under accession numbers FO9255992 to FO926003, FO681292 and KP164990 (**Supplementary Table S1**).

## Author Contributions

NC and VG designed the study and wrote the manuscript. NC, VT, TR, GM, TA, RI, AP-H, and VG contributed to data analyses. All authors read and approved the final version of the manuscript.

## Conflict of Interest Statement

The authors declare that the research was conducted in the absence of any commercial or financial relationships that could be construed as a potential conflict of interest.
