# Supplementary figures and images for: Common Bean Subtelomeres Are Hot Spots of Recombination and Favor Resistance Gene Evolution
Source: Front Plant Sci. 2018 Aug 14;9:1185. doi: 10.3389/fpls.2018.01185 (PMC6102362; doi:10.3389/fpls.2018.01185)

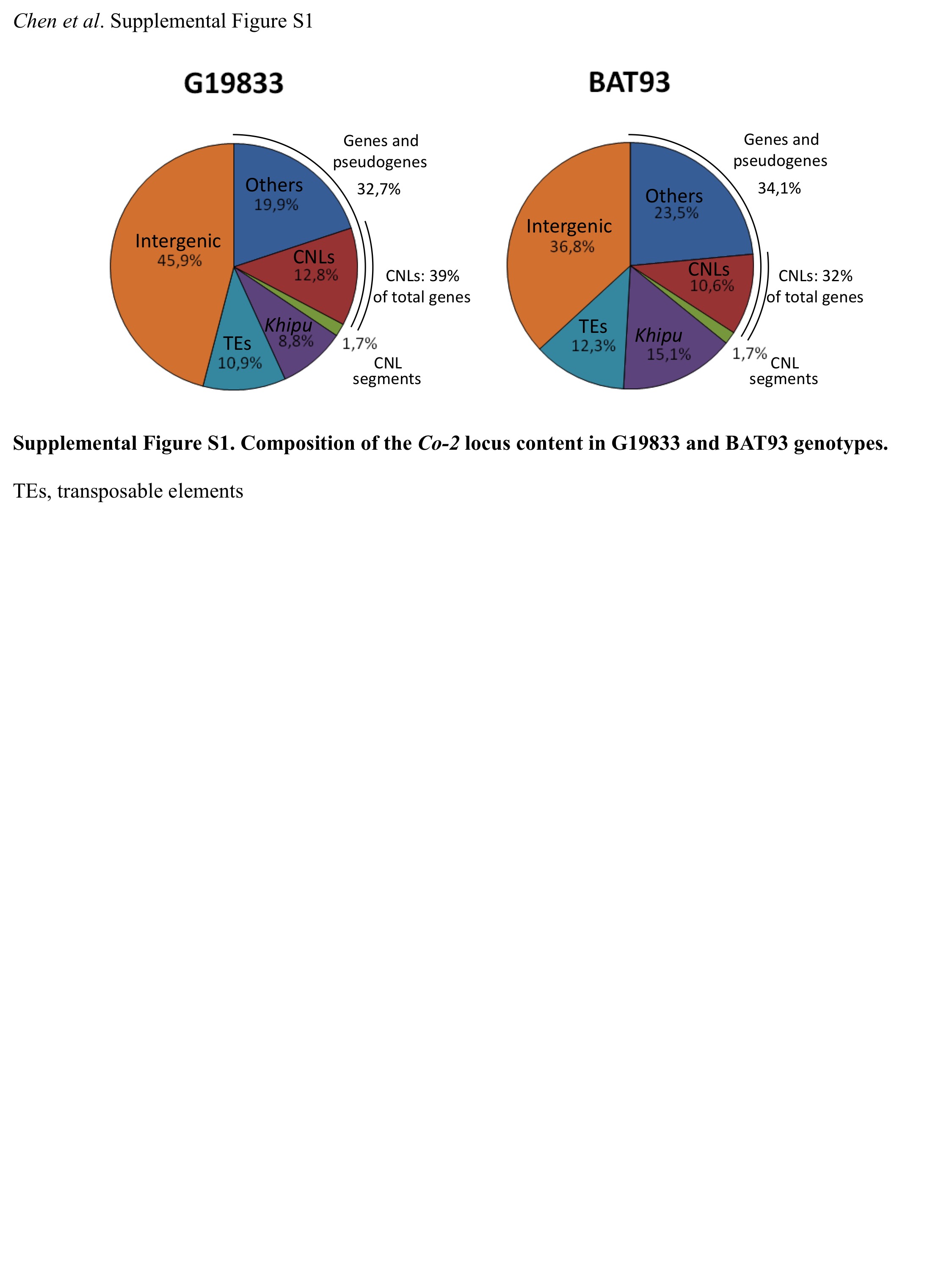

Supplement: Supplementary file 11 [file Image_1.JPEG]

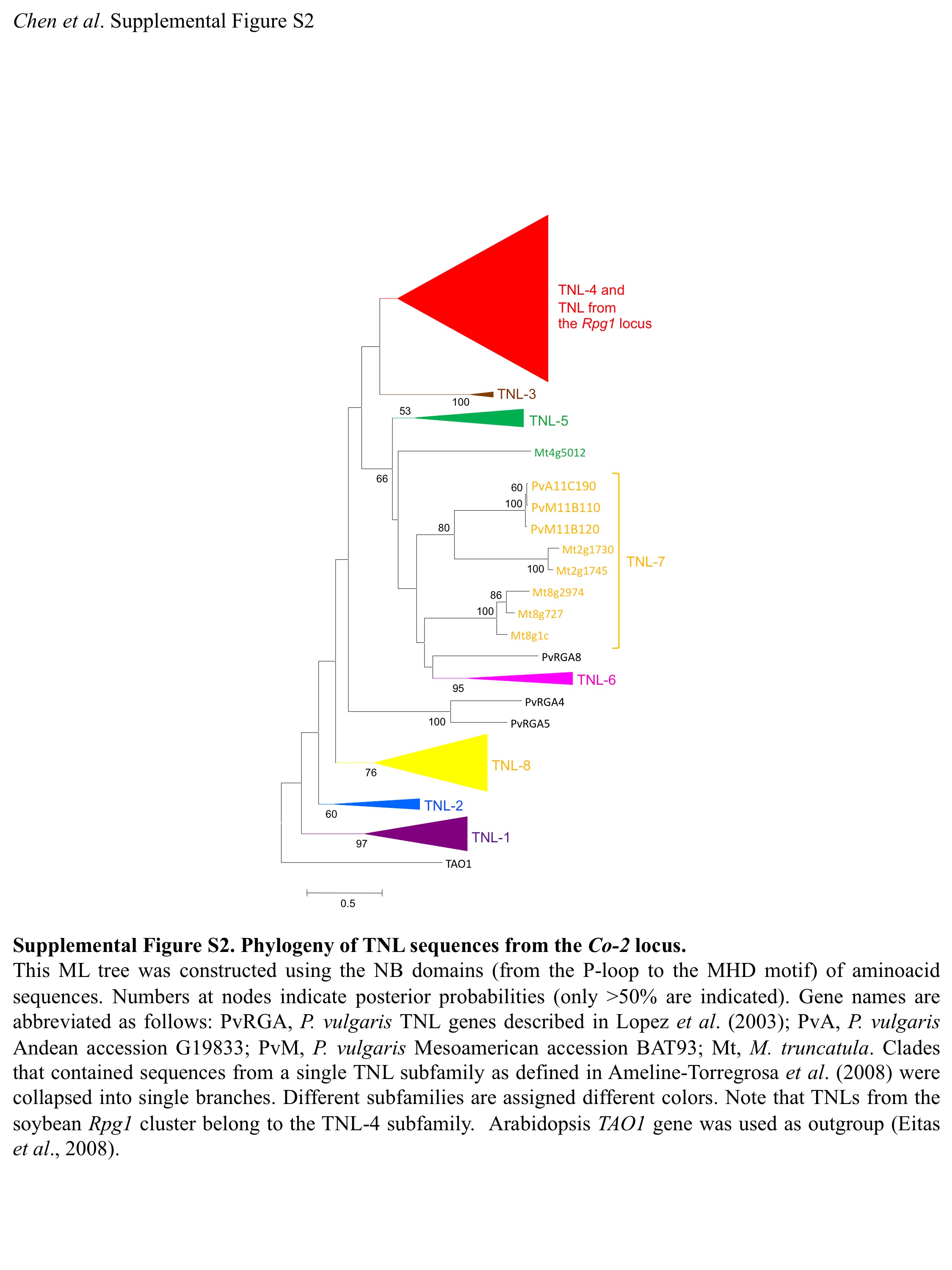

Supplement: Supplementary file 12 [file Image_2.JPEG]

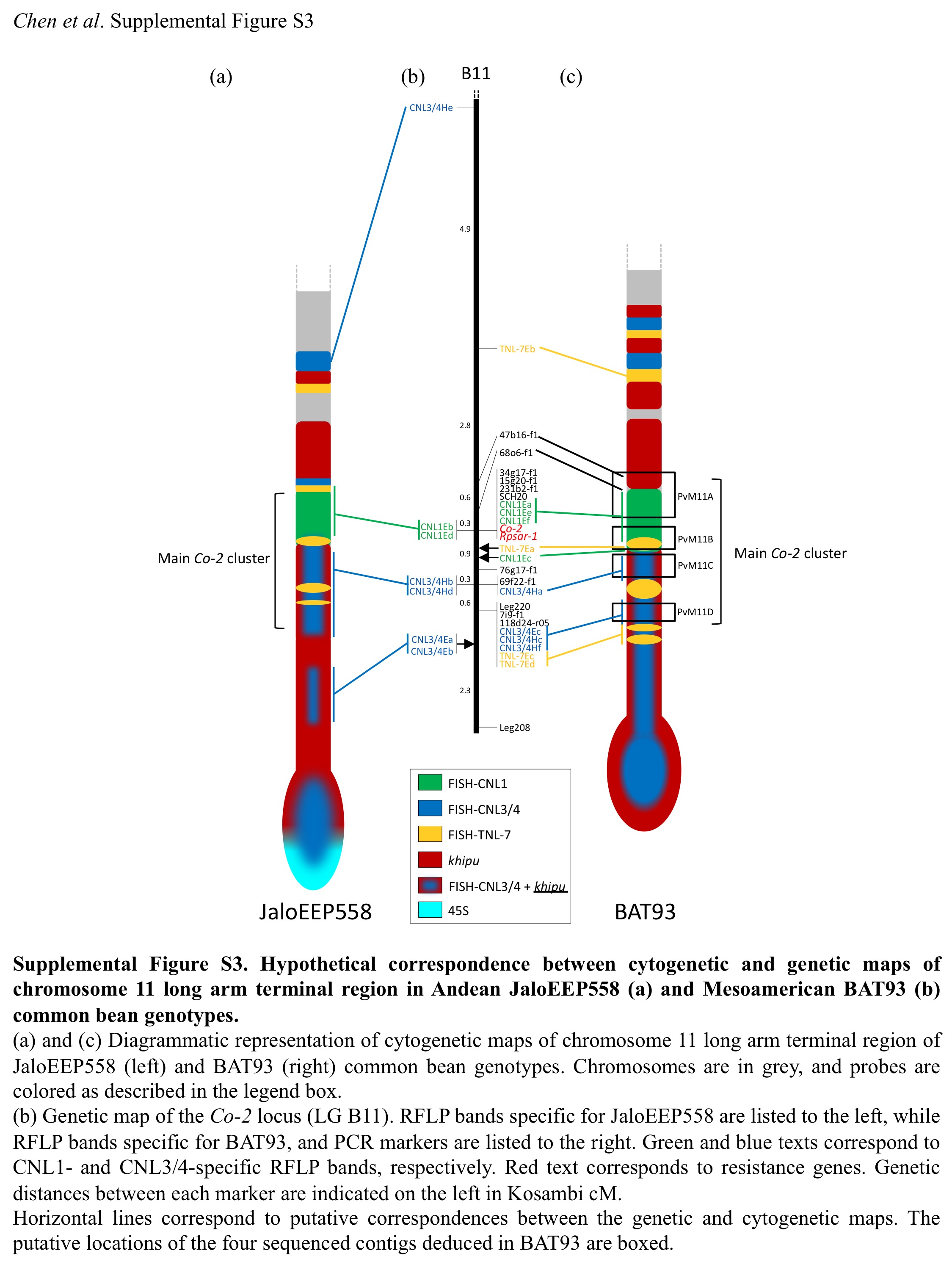

Supplement: Supplementary file 13 [file Image_3.JPEG]

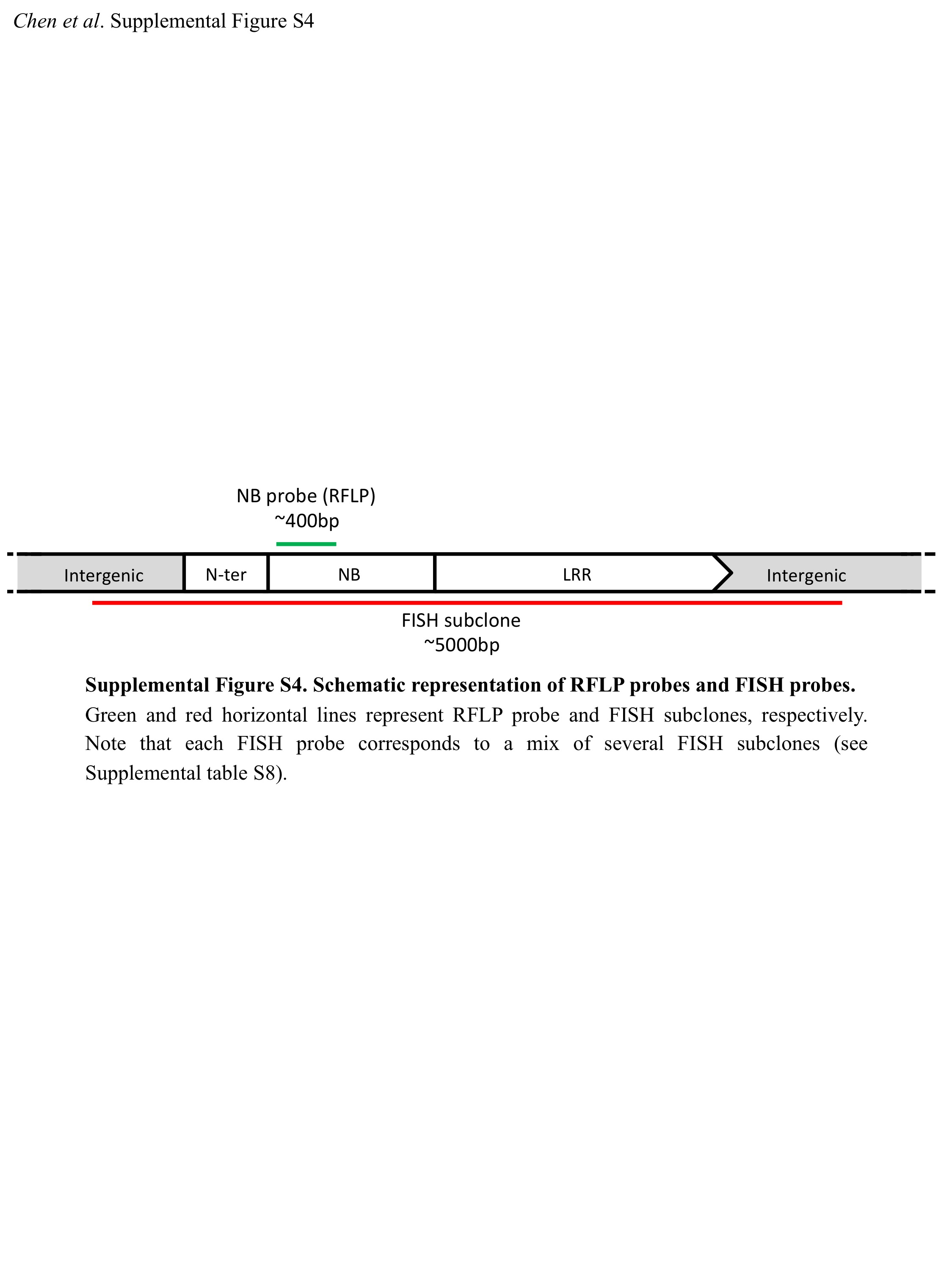

Supplement: Supplementary file 14 [file Image_4.JPEG]

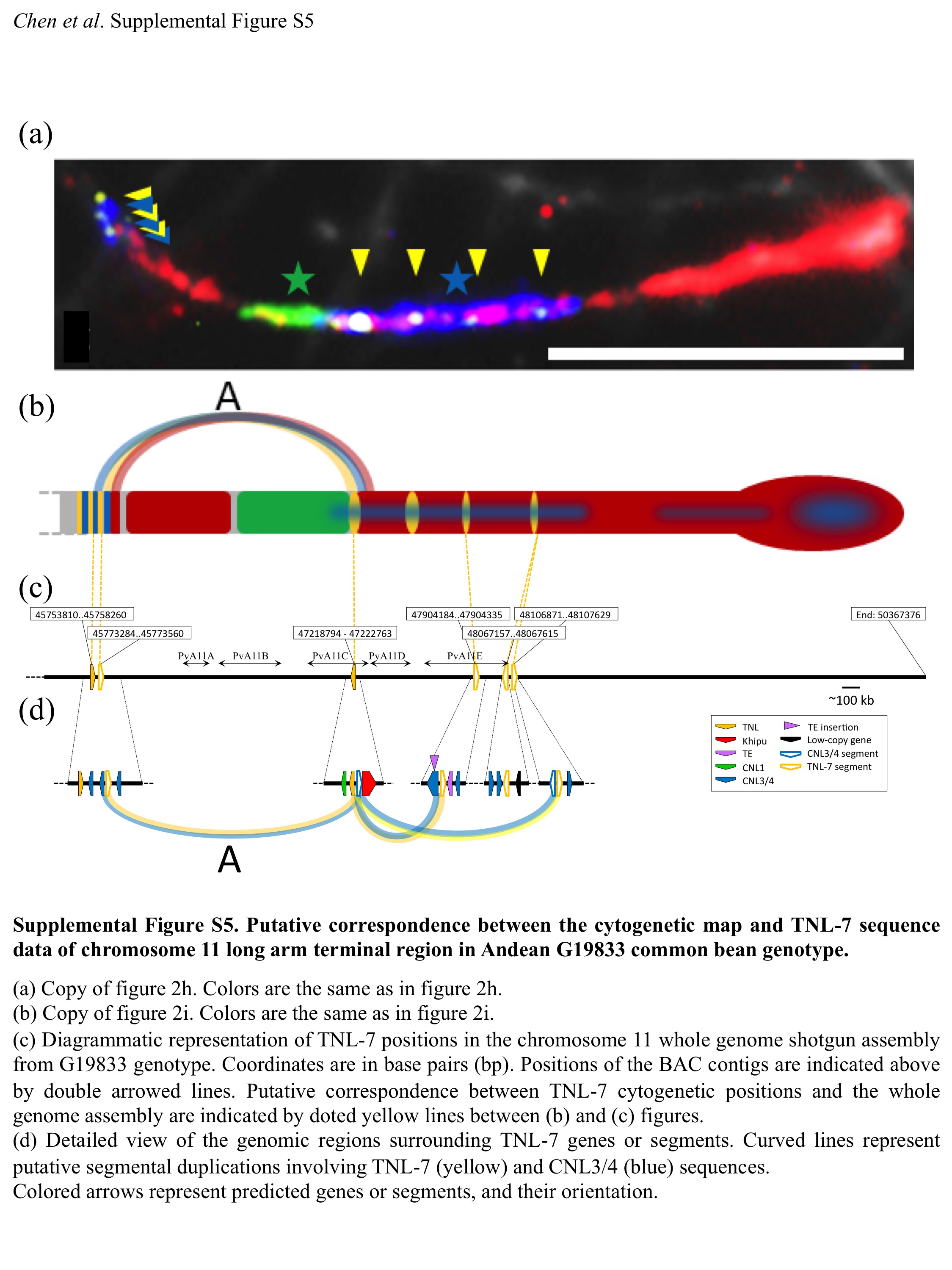

Supplement: Supplementary file 15 [file Image_5.JPEG]
